# Hemichorea-Hemiballismus as a Presentation of Cerebritis from Intracranial Toxoplasmosis and Tuberculosis

**DOI:** 10.5334/tohm.576

**Published:** 2021-01-20

**Authors:** Nico Paulo M. Dimal, Nigel Jeronimo C. Santos, Nikolai Gil D. Reyes, Mina N. Astejada, Roland Dominic G. Jamora

**Affiliations:** 1 Department of Neurosciences, College of Medicine – Philippine General Hospital, University of the Philippines Manila, PH; 2 Department of Medicine, College of Medicine – Philippine General Hospital, University of the Philippines Manila, PH

**Keywords:** hemichorea, hemiballismus, toxoplasmosis, cerebritis, intracranial tuberculosis

## Abstract

**Background:**

There is limited literature documenting hemichorea-hemiballism (HCHB) resulting from co-infection of toxoplasmosis and tuberculosis (TB) in acquired immunodeficiency syndrome (AIDS). Toxoplasmic abscess is the most common cause while TB is a rare etiology.

**Case Description:**

We describe a 24-year-old male with AIDS-related HCHB as the presentation of cerebritis on the right subthalamic nucleus and cerebral peduncle from intracranial toxoplasma and TB co-infection. Antimicrobials and symptomatic therapy were given. Marked improvement was seen on follow-up.

**Discussion:**

HCHB may be the initial presentation of intracranial involvement of this co-infection in the setting of AIDS and is potentially reversible with timely management.

**Highlights:**

- Hemichorea-hemiballismus (HCHB) may be an initial presentation of intracranial involvement of concomitant toxoplasmosis and tuberculosis causing focal cerebritis in the contralateral subthalamic nucleus and cerebral peduncle, particularly in the setting of human immunodeficiency virus infection.
- Acquired immunodeficiency syndrome-related HCHB is potentially reversible with timely diagnosis and treatment.

## 1. INTRODUCTION

The prevalence of movement disorders among human immunodeficiency virus (HIV)-infected adult population ranges from 2% to as high as 44%, with hemichorea-hemiballism (HCHB) being one of the most common presentations [1]. HCHB is a hyperkinetic movement disorder characterized by involuntary, irregular movements of variable amplitude affecting one side of the body [2]. Although the two phenomenologies share a similar pathophysiology, proximally predominant, high-amplitude movements are ascribed to ballism, while more distal, lower amplitude movements are often termed chorea [2,3]. HCHB in this population is almost invariably caused by a mature toxoplasmic abscess in the subthalamic nucleus (STN) [1,4] in a *Toxoplasma*-seropositive patient and often in the setting of established acquired immunodeficiency syndrome (AIDS) [1,4] as summarized in ***Table 1***. However, because the diagnosis of toxoplasmosis is often presumptive [31] and the imaging findings are non-specific [32,33,34], other common opportunistic infections that may cause the movement disorder must also be considered [4]. HIV-infected adults are 21–37 times more likely to acquire tuberculosis in particular [35], and those with systemic TB are five times more predisposed to have central nervous system involvement [35]. Although rare, HCHB may be secondary to intracranial TB complicated by infarctions, TB abscess or tuberculomas as seen in ***Table 1*** [25,28]. There is limited literature documenting HCHB as a result of opportunistic co-infections of toxoplasmosis and TB in HIV/AIDS patients. Here, we present the atypical case of an adult male with AIDS-related HCHB as the initial presentation of intracranial involvement from these infections.

**Table 1:** Summary of published cases* of chorea and ballism associated with cerebral toxoplasmosis and intracranial tuberculosis. Abbreviations: ART, antiretroviral therapy; BG, basal ganglia; CSF, cerebrospinal fluid; CT, computed tomography; CXR, chest radiograph; d, day/s; F, female; HB, hemiballismus; HC, hemichorea; HCHB, hemichorea-hemiballismus; HIV, human immunodeficiency virus; HR, isoniazid, rifampicin; HRZE, isoniazid, rifampicin, pyrazinamide, ethambutol; M, male; mo, month/s; MRI, magnetic resonance imaging; NR, not reported; PCR, polymerase chain reaction; PPD, purified protein derivative; Pt, patient; Pyr, pyrimethamine; STN, subthalamic nucleus; *T. gondii, Toxoplasma gondii*; TB, tuberculosis; TMP/SMX, trimethoprim/sulfamethoxazole; w, week/s; y, year/s. Legends: * Included are all relevant articles with either English full text or abstract with sufficient patient data for review; specific data is included when available. ^†^ CD4 count in cells/mL in parentheses when available. ^§^ Died due to other causes. ** Definite TB meningitis is based on autopsy, positive cerebrospinal fluid smear or culture for acid-fast bacilli while probable TB meningitis is based on clinical and CSF findings, evidence of TB in extraneural sites, positive CSF enzyme-linked immunosorbent assay or adenosine deaminase activity or if at least two of the following were positive (Mantoux test, CXR, CT or MRI, and a history of TB). ^††^ Included patients had clinical or laboratory evidence of central nervous system tuberculosis.


AUTHOR & YEAR	NO. OF CASES	AGE IN YEARS/SEX	HIV STATUS (CD4 COUNT)^†^	MODE OF ONSET AND SITE	INVESTIGATIONS	TREATMENT	CLINICAL OUTCOME	RADIOLOGIC RESPONSE

**Cerebral toxoplasmosis (*Total number of cases = 29*)**								

Navia [5] 1986	2	Pt 1: NR/M	+	Chronic, chorea of four limbs	CT: bilateral BG hypodense lesions, positive *T. gondii* serology, autopsy revealed *T. gondii* tachyzoites on multiple brain lesions	Pyr, sulfadiazine	Death	NR

		Pt 2: NR/M	+	Chronic, left choreoathetosis	CT: left internal capsule and right occipital abscess, positive *T. gondii* serology, autopsy revealed *T. gondii* tachyzoites on multiple brain lesions including BG and thalamus	Pyr, sulfadiazine	Death	NR

Nath [6] 1987	3	Pt 1: 26/F	+	NR, right HCHB	CT: left BG and frontal abscess, positive *T. gondii* titers in CSF, positive CSF cryptococcal antigen	NR	Improved	Improved

		Pt 2: 56/M	+	NR, right HB	CT: normal, positive *T. gondii* titers in CSF, autopsy showed toxoplasmosis abscess on left STN	NR	Death	NR

		Pt 3: 47/M	+	NR, right HCHB	CT: left BG and cerebral hemisphere abscess, positive *T. gondii* serology	NR	NR	NR

Dewey [7] 1989	2	Pt 1: 26/F	+	Acute, right HCHB, right face	CT or MRI: left BG or frontal lobe *Toxoplasma* abscess	Haloperidol	Partial recovery	NR

		Pt 2: 47/M	+	Acute, right HCHB	CT or MRI: left BG or cerebral hemisphere *Toxoplasma* abscess	Pyr sulfate	Partial recovery	NR

Sanchez-Ramos [8] 1989	1	33/F	+	Subacute, left HCHB	CT: right STN, thalamus, cerebellum and left caudate abscess, positive *T. gondii* serology	Pyr, sulfadiazine	Complete recovery at 2 mo^§^	Marked resolution at 20 d

Awada [9] 1993	1	33/F	+	Subacute, right HB	CT: left thalamus and STN lesion, negative *T. gondii* serology	Pyr, sulfadiazine	Complete recovery at 6 w	Complete resolution at 6 w

Nath [10] 1993	1	32/M	+	Chronic, left HCHB, left face	CT: right globus pallidus, bilateral cerebral hemisphere abscess, positive *T. gondii* serology	Pyr, sulfadiazine, haloperidol	Partial recovery at 10 d^§^	Partial resolution at 10 d

Garretto [11] 1995	1	26/M	+ (218)	Subacute, left HCHB	MRI: right cerebral peduncle, frontal and left temporo-occipital abscess, positive *T. gondii* serology	Pyr, sulfadiazine, haloperidol	Minimal recovery	Partial resolution after 6 w

Manji [12] 1995	1	28/F	+	Subacute, right choreoathetosis	CT: bilateral lentiform nuclei, thalami, cerebral hemisphere lesions, positive *T. gondii* serology	Pyr, sulfadiazine	Partial recovery at 5 w	Complete resolution at 5 mo

Krauss [13] 1996	1	32/M	+	Subacute, right HB	CT or MRI: abscess in left STN from toxoplasmosis	Antitoxoplasmosis; underwent cervical ventrolateral chordotomy	No recovery with medication; complete recovery after surgery at 6 mo	Complete resolution

Maggi [14] 1996	3	Pt 1: 27/M	+ (50)	NR, left arm choreoathetosis	MRI: right thalamic, bilateral hemisphere abscess from toxoplasmosis	Pyr, sulfadiazine	Complete recovery at 1 mo	NR

		Pt 2: 31/M	+ (30)	NR, left HCHB	MRI: right caudate, STN abscess from toxoplasmosis	Pyr, sulfadiazine	Complete recovery at 1 mo	Complete resolution at 4 mo

		Pt 3: 32/M	+ (30)	NR, left choreoathetosis	MRI: right STN, midbrain and bilateral hemisphere abscess from toxoplasmosis	Pyr, sulfadiazine	Complete recovery at 10 d	Complete resolution at 3 mo

Piccolo [15] 1999	2	Pt 1: 27/M	+	Acute, right HC	CT and MRI: abscess in left STN, positive *T. gondii* serology	Pyr and sulfamethopyrazine	Complete recovery at 2 mo	Complete resolution at 1 mo

		Pt 2: 35/M	+ (10)	Acute, chorea of four limbs, face and mouth	MRI: abscess in right cerebral peduncle and BG, left occipital, temporal thalamocapsular and frontal operculum, autopsy revealed *T. gondii* tachyzoites on circular brain lesions	NR	Death	NR

De Mattos [16] 2002	6	Mean age 32.5 (Range: 27 to 40)/all male	+	Acute, left HCHB in 5 cases, right HCHB in 1 case	Ring enhancing lesions in striatum or in the frontal lobe suggestive of toxoplasmosis	NR	NR	NR

Piccolo [17] 2003	2	Pt 1: 27/NR	+	NR, hemichorea	Toxoplasma abscess in contralateral STN	NR	Complete recovery at 6 mo	NR

		Pt 2: 35/NR	+	NR, generalized chorea	Pathologically confirmed toxoplasmosis in BG	NR	Death	NR

Rabhi [18] 2011	1	59/F	+ (91)	Chronic, left HCHB	MRI: right capsulothalamic abscess, positive *T. gondii* serology	TMP/SMX	Complete recovery at 2 w	Marked resolution

Sta. Maria [19] 2012	1	30/M	+	Acute, left HC	MRI: Abscess in cerebral peduncle and frontal lobe, positive *T. gondii* serology	TMP/SMX	NR	NR

Moccia [20] 2013	1	32/F	+ (250)	Subacute, right HCHB	MRI: ring enhancing lesion in left caudate to midbrain, *T. gondii* IgG positive 25 UI/mL	Pyr, sulfadiazine, haloperidol, ART	Complete recovery at 3 mo	Small residual lesion in the midbrain at 3 mo

**Intracranial tuberculosis *(Total number of cases = 23)***								

Bedwell [21] 1960	1	39/M	NR	Subacute, left HC	Autopsy revealed tuberculoma in the right STN	Anti-TB	Death	NR

Riela [22] 1982	1	11 mo/M	NR	Subacute, left choreoathetosis	CT: hypodensity in right BG, positive *M. tuberculosis* CSF culture, positive PPD	HR	Complete recovery	NR

Delaporte [23] 1983	1	33/NR	NR	NR, left HB	CT: tuberculoma in right STN	Anti-TB	Complete recovery at 1 y	Marked resolution at 1 y

Babikian [24] 1985	1	2.5/M	NR	Acute, right HCHB	CT: bilateral BG infarct, hydrocephalus, positive *M. tuberculosis* CSF culture	HR, streptomycin, diphenhydramine	Partial recovery at 1 y	NR

Alarcón [25] 2000	7**	Range: 0.5 to 27/4 male, 3 female	NR	Acute to subacute, 3 HC, 4 generalized chorea	5 cases of definite TB meningitis, 2 probable TB meningitis, HC cases had infarcts in contralateral thalamus, caudate or internal capsule; generalized chorea cases had cortico-subcortical atrophy, hydrocephalus or bilateral caudo-capsular infarct	Anti-TB	2 with complete recovery, 3 with partial recovery, 2 died at 2 y	NR

Kalita [26] 2003	1	16/F	– (191)	Subacute, left HC, left face	CT: tuberculoma in right caudate and putamen, hydrocephalus and meningeal enhancement, positive CSF acid-fast bacillus smear, right upper fibrocavitary lesion on CXR	HRZE	Marked improvement at 1 w, complete recovery at 3 mo	NR

Ozer [27] 2006	1	78/M	–	Chronic, right HCHB	MRI: tuberculoma in the left thalamus, STN and midbrain, positive PPD	HRZE	Partial recovery at 6 mo, complete recovery at 2 y	Partial resolution at 6 mo

Alarcón [28] 2011	8^††^	Pt 1: 9/F	NR	Subacute, left HC	CT or MRI: tuberculoma in frontal region and right thalamus, hydrocephalus	Anti-TB	Complete recovery at 1 y	NR

		Pt 2: 16/F	NR	Subacute, generalized chorea	CT or MRI: tuberculoma in left thalamus, hydrocephalus	Anti-TB	Complete recovery at 1 y	NR

		Pt 3: 28/F	NR	Subacute, right HC	CT or MRI: tuberculoma in left thalamus and frontoparietal region, hydrocephalus	Anti-TB	Complete recovery at 1 y	NR

		Pt 4: 22/M	NR	Subacute, right HC	CT or MRI: tuberculoma in left caudate and thalamus, hydrocephalus	Anti-TB	Partial recovery at 1 y	NR

		Pt 5: 8/F	NR	Subacute, right HC	CT or MRI: tuberculoma in left thalamus and parietal region, right cerebellum	Anti-TB	Complete recovery at 1 y	NR

		Pt 6: 17/F	NR	Subacute, right arm chorea	CT or MRI: left frontotemporal tuberculoma	Anti-TB	Partial recovery at 1 y	NR

		Pt 7: 42/F	NR	Subacute, right arm chorea	CT or MRI: right cerebellar tuberculoma	Anti-TB	Death	NR

		Pt 8: 27/F	NR	Subacute, right HC	CT or MRI: tuberculoma in left frontotemporal region and thalamus	Anti-TB	Partial recovery at 1 y	NR

Pinto [29] 2013	1	14/F	–	Acute, right HC	MRI: left caudate and lenticulo-capsular infarct, diagnosed miliary TB (meningitis and pulmonary disease)	HRZE	NR	NR

Rubio-Hernandez [30] 2020	1	33/F	–	Subacute, left HCHB	MRI: multiple tuberculomas in right thalamus, STN, cerebral peduncle, cerebellum and subcortical white matter, positive CSF *M. tuberculosis* PCR, CXR showed miliary pattern and left nodular lesion	HRZE, olanzapine	Complete resolution at 6 mo	Partial resolution at 1 mo


## 2. CASE DESCRIPTION

A 24-year-old man presented in our emergency department with a three-day history of involuntary movements of the left extremities. These were described as non-suppressible, mildly disabling, relieved by sleep, and rapidly progressing in frequency and amplitude. Few hours prior to admission, the movements became more violent and involved his left lower limb making ambulation difficult. There were no changes in sensorium nor other focal symptoms. He was diagnosed two months earlier with HIV infection after presenting with weight loss, fever and draining cervical lymph nodes. Lymph node biopsy revealed positive acid-fast bacillus (AFB) smear while his chest radiograph was consistent with pulmonary TB, for which he was started on isoniazid, rifampicin, pyrazinamide and ethambutol (HRZE). His family, medication and toxicologic history were unremarkable. On examination, he was cachectic, tachycardic, normotensive and afebrile. He had multiple, non-tender right cervical lymph nodes with scanty serosanguinous discharge. He was awake and conversant, but had subtle difficulty following complex commands. Funduscopic examination was normal with no evidence of Kayser-Fleischer rings. Cranial nerve examination did not show any abnormalities nor facial involuntary movements. He had random, purposeless, dance-like, intermittently flinging movements of the left extremities that resulted in shrugging and internal rotation of the shoulder, as well as random flexion-extension and pronation-supination of the distal upper limb. His gait was interrupted by sudden, irregular internal rotation of the hip, flexion-extension of the knee, and inversion-eversion of the foot (***Video*, Segment 1**). These movements were consistent with left HCHB. His limbs were normotonic with 4/5 motor strength on the left extremities. The rest of the examination was normal.

**Video:** **Hemichorea-hemiballismus (HCHB). Segment 1.** Patient at presentation three days after symptom onset showing random and occasionally ballistic movements of the left upper and lower limbs. **Segment 2.** Post-treatment at nine months after symptom onset showing marked reduction in amplitude and intensity of the HCHB and residual mild choreoathetoid movements of the left upper limb.

**Figure 1:**
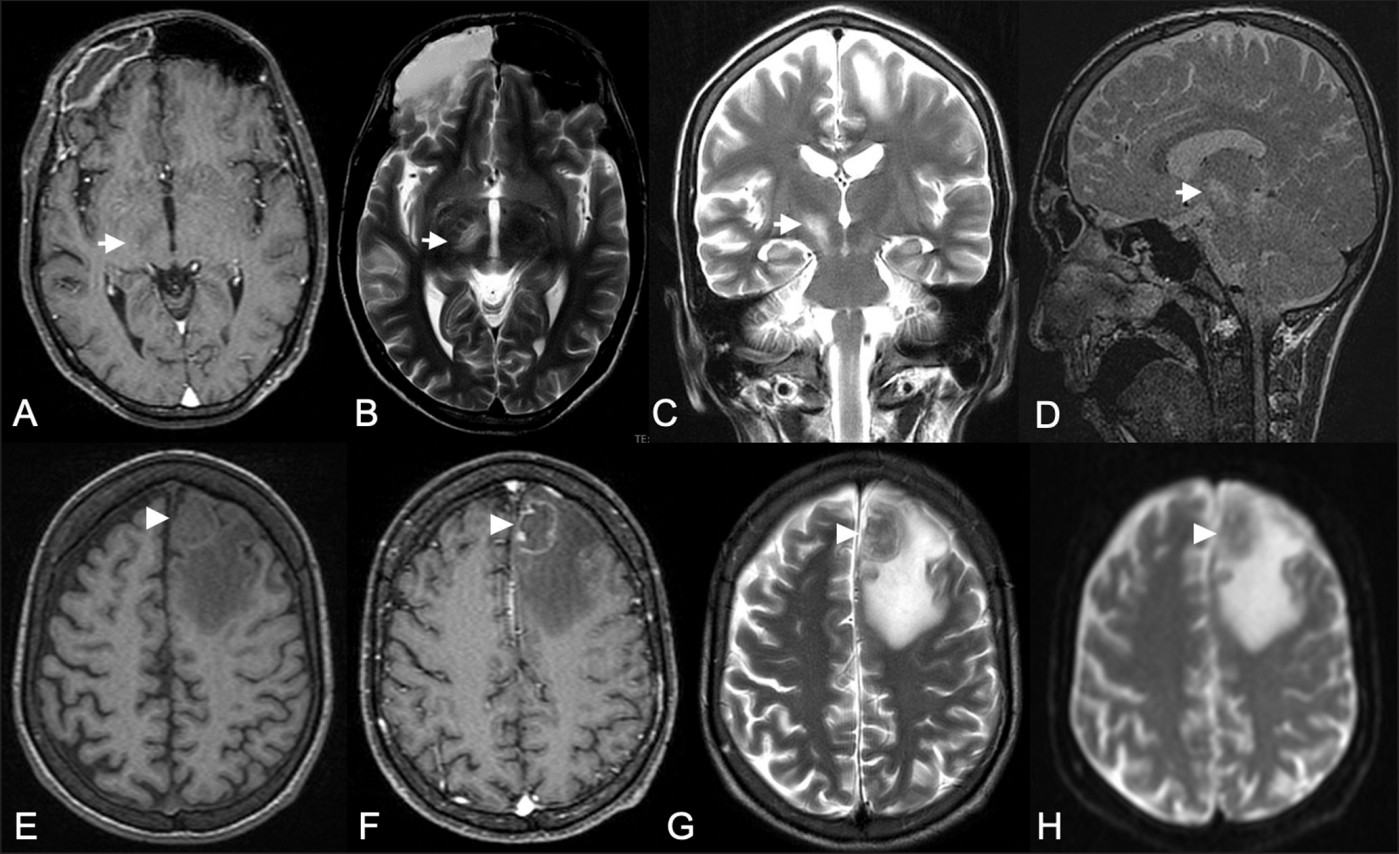
**Cranial magnetic resonance imaging of the patient at presentation.** A non-enhancing hypointense focus (white arrow) on postcontrast T1 weighted imaging **(A)** is seen on the right subthalamic region and cerebral peduncle appearing hyperintense on axial **(B)**, coronal **(C)** and sagittal **(D)** T2 weighted images, representing focal cerebritis. Also seen is an abscess formation in the left frontal region (white arrowhead) with a slightly hyperintense rim on noncontrast T1 weighted image **(E)** suggestive of tuberculoma. On postcontrast axial T1 weighted image **(F)**, it appears rim-enhancing with a nodular area of enhancement along its medial wall possibly an “eccentric target sign”, with a hypointense core and surrounding moderate vasogenic edema on axial T2 weighted **(G)** and diffusion weighted **(H)** imaging. Considerations for this space-occupying lesion include a tuberculoma and cerebral toxoplasmosis.

On laboratory testing, he had leukopenia with a peripheral white blood cell count of 4.2 × 10^9^/L and a peripheral blood smear showing slight poikilocytosis with no acanthocytes. He had elevated erythrocyte sedimentation rate at 121 mm/hour (normal values [NV]: 0–15) and C-reactive protein at >12 mg/L (NV: <6). He had mild hyponatremia with a sodium of 133 mmol/L (NV: 137–145 mmol/L), while his random blood sugar, liver, kidney and thyroid function tests were normal. His chest radiograph revealed bilateral apical nodular infiltrates, while abdominal imaging was unremarkable. Given the aforementioned past history, we investigated for possible infectious etiologies of the patient’s HCHB. He had a positive HIV Western blot test assay and a CD4 count of 92/mL on admission. His serum toxoplasma IgG was markedly elevated at >300 IU/mL (NV: <8) while syphilis and anti-streptolysin O (ASO) titers were negative. Cranial magnetic resonance imaging (MRI) revealed a focal cerebritis in the right STN and right cerebral peduncle (***Figure 1, A–D***) as well as a left frontal abscess formation (***Figure 1, E–H***). A lumbar puncture was performed with an opening pressure of 20 cmH_2_0 (NV: 8–18 cmH_2_0). His cerebrospinal fluid (CSF) was acellular with protein at 89 mg/dL (NV: 12–60) and glucose at 45.45 mg/dL (NV: 40–70), the latter being 29% of the serum level. CSF TB polymerase chain reaction (PCR) was positive while CSF culture for *M. tuberculosis* had no growth. CSF AFB smear, cryptococcal antigen, bacterial and fungal cultures were all negative.

**Figure 2:**
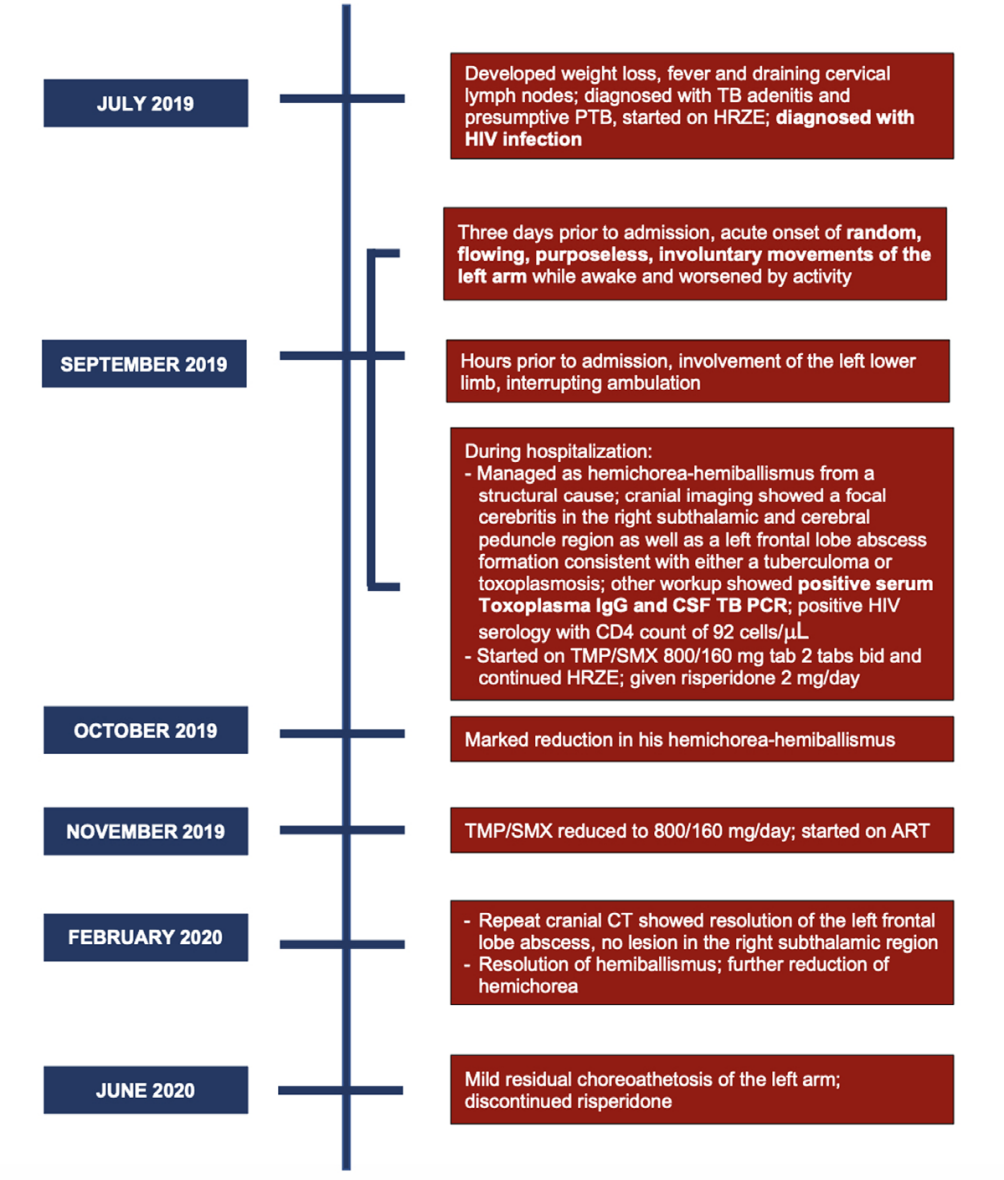
**Timeline of events.** This timeline highlights the chronology of the patient’s symptoms, work-up, management, and status on follow-up. Abbreviations: ART, antiretroviral therapy; CSF, cerebrospinal fluid; CT, computed tomography; HIV, human immunodeficiency virus; HRZE, isoniazid/rifampicin/pyrazinamide/ethambutol; PCR, polymerase chain reaction; PTB, pulmonary tuberculosis; TMP/SMX, trimethoprim/sulfamethoxazole.

With these clinicoradiologic findings, we managed him as a case of acute left HCHB secondary to a right focal cerebritis from concurrent presumptive cerebral toxoplasmosis and definite intracranial tuberculosis. He was immediately started on oral trimethoprim/sulfamethoxazole (TMP/SMX) for the toxoplasmosis. His anti-TB regimen consisted of HRZE for two months and isoniazid/rifampicin for 10 months. To address his ballistic movements, he was given risperidone which was titrated up to 2 mg/day. His HCHB were most evident during his waking hours and remained of moderate severity, necessitating measures to avoid limb injury. He was discharged after three weeks on the previously described treatment regimen, with minimal change in his HCHB. At one-month follow-up, we noted significant reduction in his HCHB. Due to the evident clinical improvement, TMP/SMX dose was reduced at two months after discharge. Antiretroviral therapy was started during this time, while his anti-TB medications were continued. A surveillance cranial contrast-enhanced computed tomography (CT) was done after four months of treatment demonstrating complete resolution of his cerebral lesions. At ninth-month follow-up, there were only occasional mild, non-disabling choreoathetoid movements of the left arm (***Video*, Segment 2**), allowing discontinuation of risperidone. ***Figure 2*** summarizes the timeline of events and the management done.

## 3. DISCUSSION

Our case affirms the characteristics of HCHB among reported cases of chorea-ballism in HIV-infected adults [1,4] namely, the (1) presence of an identified structural pathology in the contralateral STN and midbrain believed to cause the chorea, (2) the hemidistribution, (3) multiplicity of the lesions, (4) acute onset [1], (5) lack of facial involvement which has only been seldom reported [7,10,15] and finally, (6) the eventual clinical and radiologic improvement seen [1,4].

To our knowledge, our case is the first to document HCHB associated with cerebral toxoplasmosis that is in conjunction with intracranial TB infection occurring in the setting of HIV infection [1,10]. The HCHB in this case heralded the presence of AIDS-defining opportunistic infections, which is a distinct stage of HIV disease [36] complicated by neurological abnormalities in as high as 60% of patients [4,37]. In patients with movement disorders associated with AIDS, identifying the opportunistic infection is the initial goal of management [4]. When HCHB occurs in this setting, a singular infectious etiology, primarily toxoplasmosis, is more commonly pursued which if negative, prompts consideration of alternative causes [1,4]. However, our case illustrates that in the appropriate clinical context, more than one intracranial infection is possible and should be sought. In contrast to published literature [1,4], HCHB in our case was from a cerebritis that may represent a beginning tuberculoma or a toxoplasmic abscess. Cerebritis is a nondescript area of parenchymal inflammation due to altered vascular permeability that heralds the development of an abscess and is seen on MRI as an area of hyperintensity on T2 weighted imaging, often with patchy contrast enhancement [38,39]. This is already a known early stage of toxoplasmic encephalitis [31] although other factors in our case led to the presumptive diagnosis of cerebral toxoplasmosis, namely, the presence of HCHB, multifocality of the brain lesions, *T. gondii* seropositivity and low CD4 count [31]. On the other hand, although TB cerebritis is very rare [39], the positive CSF TB PCR [35] and the MRI characteristics of the left frontal lobe lesion corroborated its tuberculous nature [40,41]. The rarity of TB cerebritis may be attributed to its delayed clinical presentation, which typically only manifests when mass effect ensues [42]. This case also demonstrates that a deep location as well as the presence of multiple focal tuberculous lesions may influence the appearance of movement disorders as suggested in Alarcón’s cohort of 49 patients with tuberculoma [28]. Although we believe that both infections were contributory to the HCHB at the time of presentation, one limitation encountered was the lack of baseline neuroimaging, CD4 count and HIV viral load at the time of initial HIV and TB diagnosis, which could have helped elucidate the temporality of these infections in relation to HCHB.

Literature on concomitant intracranial co-infections in AIDS patients is limited, with a frequency of coexistent neurological diseases in this population ranging from 9% to 24% [31]. Currently, there are no studies describing their interplay as an underlying mechanism for HCHB, whether in the presence or absence of HIV infection. Acute disruption and altered firing rate of the STN and its efferent pathways due to a destructive focus, in addition to the direct effects of HIV on the basal ganglia circuitry, possibly contribute to the pathogenesis of chorea-ballism in this population [2,20,27,28,37,43]. As is usually the case among AIDS patients, multiple conditions may affect a portion of the nervous system simultaneously [36] and it is imperative to address these with adequate antimicrobial therapy.

The most effective treatment [4] in this case of co-infection remains to be trimethoprim/sulfamethoxazole [31], which is locally available, as well as HRZE [35]. The subsequent treatment response obviated the need for biopsy [31,44]. While we could not entirely discount its benefit in the acute phase, long-term symptomatic therapy with risperidone was no longer necessary in our case as this is commonly reserved for those with persistent or disabling symptoms [4,37]. Although response of chorea-ballism to antitoxoplasmosis and anti-TB therapy is variable [1,4,20], the marked improvement seen in our patient may attest to the reversible nature of AIDS-related HCHB, as is often seen after treatment [4]. Other factors that may affect overall prognosis of intracranial toxoplasmosis and TB include timing of diagnosis and therapy, clinical stage of the disease, and progression of neurological involvement [20,31,35].

In conclusion, this case demonstrates that HCHB may be the initial presentation of intracranial involvement of concomitant toxoplasmosis and TB in the setting of HIV infection. AIDS-related HCHB is potentially reversible with timely diagnosis and treatment.
